# Unraveling the Link Between Trauma and Problematic Alcohol Use Over Time: The Influence of Executive Function and Social Cognition Deficits

**DOI:** 10.1002/cpp.70169

**Published:** 2025-11-06

**Authors:** Yi‐Ying Lu, Ming‐Hong Hsieh, Triantoro Safaria, Yu‐Lien Huang

**Affiliations:** ^1^ Department of Psychology Chung Shan Medical University Taichung Taiwan; ^2^ Department of Psychiatry Chung Shan Medical University Hospital Taichung Taiwan; ^3^ School of Medicine Chung Shan Medical University Taichung Taiwan; ^4^ Department of Magister of Professional Psychology, Faculty of Psychology Universitas Ahmad Dahlan Jogjakarta Indonesia; ^5^ Clinical Psychological Room Chung Shan Medical University Hospital Taichung

**Keywords:** emotion recognition, executive function, posttraumatic symptoms, problematic alcohol use, theory of mind

## Abstract

The co‐occurrence of posttraumatic stress symptoms (PTSS) and problematic alcohol use (PAU) is well established, yet the mechanisms linking them remain unclear. Trauma may impair executive function (EF) and social cognition, such as emotion recognition and theory of mind (ToM), which hinder emotional regulation and increase reliance on alcohol as a coping strategy. This study examined whether deficits in EF and social cognition mediate the relationship between PTSS and PAU. A two‐wave longitudinal design was employed with 200 Taiwanese adults exposed to trauma recruited from the community and psychiatric clinics. At baseline (Time 1), participants completed self‐report measures of trauma, PTSS and PAU, along with computerized tasks assessing EF, emotion recognition and ToM. One month later (Time 2), PTSS and PAU were reassessed in 143 participants. PTSS at Time 1 directly predicted PAU at both Time 1 and Time 2, supporting the self‐medication hypothesis. In addition, PTSS indirectly influenced PAU through deficits in EF, emotion recognition and ToM. These findings suggest that cognitive and social‐cognitive impairments are key risk factors for alcohol misuse among trauma‐exposed individuals. Interventions targeting EF and social cognition may therefore help reduce PAU and support trauma recovery.

## Summary

- Posttraumatic stress symptoms are associated with problematic alcohol use, supporting the self‐medication hypothesis.
- Trauma can impair executive function and social cognition, including emotion recognition and theory of mind.
- These cognitive and emotional deficits may increase vulnerability to alcohol misuse among trauma‐exposed individuals.
- Interventions targeting executive function and social cognition could help reduce problematic alcohol use in this population.

## 1 Introduction

Exposure to traumatic events often leads to posttraumatic stress symptoms (PTSS) or posttraumatic stress disorder (PTSD) and may trigger self‐medication behaviours. The self‐medication hypothesis (Khantzian 1997) posits that individuals experiencing psychological distress use substances such as alcohol to alleviate negative emotions. In trauma contexts, alcohol may provide short‐term relief but ultimately reinforces a cycle of negative reinforcement that sustains both PTSS and alcohol misuse (Hawn et al. 2020).

The co‐occurrence of PTSS and problematic alcohol use (PAU) is well documented, with prevalence estimates ranging from 10% to 75% (Baker et al. 2009; Pietrzak et al. 2011). PTSD is frequently comorbid with alcohol use disorders (AUD) and dependence (Allen et al. 2016; Blanco et al. 2013; Goldstein et al. 2017) and is consistently associated with increased alcohol use and related problems over time (Grant et al. 2015; Kaysen et al. 2011; Leeies et al. 2010). Despite this strong association, the mechanisms underlying the PTSD–PAU comorbidity remain insufficiently understood.

According to the shared‐liability model (Subbie‐Saenz de Viteri et al. 2020), alcohol‐related harm following trauma arises from multiple interacting vulnerabilities rather than individual choice alone. PTSS and PAU share common risk factors, particularly deficits in executive functioning (EF), such as working memory, cognitive flexibility and inhibitory control (Day et al. 2015; Gilbertson et al. 2001), and impairments in social cognition, including difficulties in recognizing and interpreting others' emotions and intentions (Poljac et al. 2011) as well as deficits in theory of mind and emotion recognition (Maurage et al. 2011).

No study has directly examined whether combined deficits in EF and social cognition amplify the risk of PAU among trauma‐exposed individuals. To address this gap, the present study investigates how EF and social cognition interact in the relationship between PTSS and PAU. A two‐wave longitudinal design was employed to capture temporal associations between PTSS and alcohol‐related outcomes, allowing stronger inferences about the directionality of these effects.

Evidence suggests that trauma exposure is linked to deficits in EFs, including working memory, inhibitory control and cognitive flexibility (Aupperle et al. 2012). Such neurocognitive impairments have been observed in both children and adults with trauma histories (Schuitevoerder et al. 2013; Scott et al. 2015) and may increase vulnerability to addictive behaviours, such as problematic alcohol use (Silveira et al. 2020). Trauma is also associated with social cognition difficulties, including reduced emotional understanding (Shipman et al. 2000), delayed development of theory of mind (ToM) (Cicchetti et al. 2003; O'Reilly and Peterson 2015), and impairments in mentalization (Ensink et al. 2015; Huang et al. 2020). Taken together, these findings highlight how trauma disrupts both EF and social cognition, which in turn may heighten risk for maladaptive coping strategies such as alcohol misuse.

Similar impairments are consistently observed in individuals with PAU. Research shows that alcohol dependence is associated with a wide range of cognitive deficits, including slowed processing, learning difficulties, problem‐solving deficits and reduced visuospatial abilities (Ellis and Oscar‐Berman 1989; Evert and Oscar‐Berman 1995). Broader impairments in IQ, verbal fluency, processing speed, working memory, attention and memory have also been documented, with some persisting up to a year after detoxification (Stavro et al. 2013). In addition, PAU is strongly linked to social cognition deficits, particularly in emotion recognition and ToM (Bora and Zorlu 2017; Le Berre 2019). For instance, Freeman et al. (2018) found that individuals with alcohol use disorders frequently misattributed neutral facial expressions as anger or disgust, while Donadon and Osório (2017) reported reduced accuracy in recognizing fear and disgust. A meta‐analysis by Le Berre (2019) further confirmed that recently detoxified individuals exhibit marked emotional and social cognitive impairments, including difficulties in emotion expression, recognition and ToM. These findings underscore the robust association between PAU and deficits in EF and social cognition.

Despite these findings, little is known about the potential interaction between EF deficits and social‐cognitive impairments in shaping the pathway from PTSS to PAU. Recent evidence suggests that EF and social cognition, such as emotion recognition and ToM, are interrelated (Blair and Razza 2007; Lertladaluck and Moriguchi 2024). Studies have shown a positive association between neurocognition and social cognition in both healthy individuals and patients with schizophrenia (Deckler et al. 2018). Notably, EF shows a robust association with emotion recognition, and emotion recognition subsequently predicts ToM among patients with schizophrenia (Ku and Lin 2020; Huang et al. 2023). Additionally, research highlights a direct effect of cognitive function and emotion recognition in predicting ToM (Meinhardt‐Injac et al. 2018; Ku and Lin 2020). This suggests a sequential link in which EF affects emotion recognition, and that in turn predicts ToM.

Building on this foundation, the present study investigated whether executive function (EF) influences emotion recognition and theory of mind (ToM) and examined their roles in the relationship between posttraumatic stress symptoms (PTSS) and problematic alcohol use (PAU). To date, no prospective study has tested whether combined deficits in EF and social cognition amplify the risk of PAU among trauma‐exposed individuals. Addressing this gap is essential for advancing mechanistic models of the PTSS–PAU association and for developing interventions that strengthen both cognitive and social‐cognitive functioning.

## 2 Purpose of the Present Study

The impairments of EF and social cognition (emotion recognition and ToM) may contribute to the development of the co‐occurrence of PTSS and PAU, but the influence of the combination of EF deficits and social cognition impairments is lacking in the link from PTSS to PAU. In addition, a two‐wave design allows for examining the temporal association between PTSS and PAU. Thus, this study examines the potential mediating roles of EF, emotion recognition, cognitive ToM and affective ToM in the relationship between PTSS and PAU over time. The hypotheses proposed are as follows: (1) PTSS is positively associated with PAU across time, (2) EF deficits and social cognition impairments are positively associated with PTSS and PAU, and (3) EF deficits and social cognition impairments sequentially mediate the relationship between PTSS and PAU.

## 3 Method

### 3.1 Procedure

This study was approved by the Research Review Committee of a private hospital (CS2‐23083). All participation was entirely voluntary, and withdrawal at any time carried no penalty. After obtaining signed informed consent from the participants, all completed the Life Event Checklist for DSM‐5 (LEC‐5; Gray et al. 2004; Weathers et al. 2013) to assess the most distressing traumatic event they had experienced. Then, they completed the questionnaire to assess the severity of posttraumatic symptoms and alcohol usage behaviour at baseline assessment (Time 1, T1). The Wisconsin Card Sorting Test (WCST) and the Taiwan version of the A Movie for the Assessment of Social Cognition (MASC‐TW) were administered to assess their executive function and ToM. Participants' facial and prosodic emotion recognition skills were evaluated using a computerized facial and vocal expression subtest from the Taiwanese version of the Diagnostic Analysis of Nonverbal Accuracy 2 (DANVA‐2‐TW). All participants were invited to assess their posttraumatic symptoms and alcohol usage behaviour at one‐month follow‐up (Time 2, T2); each participant received a base payment rate of US$8 per hour as compensation for their participation.

Of the 200 traumatized individuals who completed the initial assessment (time 1; T1), 143 responded to the follow‐up invitation and completed the reassessment at one month (time 2; T2). The remaining 57 participants did not respond to the follow‐up invitation. A possible explanation is that, due to privacy considerations, participants had only provided an email address as the sole means of contact at T1, making email invitations more likely to be overlooked. Alternatively, some participants may have been too busy or simply forgotten to complete the reassessment.

### 3.2 Participants

The sample size is determined as a function of an alpha level of 0.05 and power at 0.80 using the statistical software G*power 3.1, resulting in 125 participants. A total of 200 trauma‐exposed adults (65.5% women, aged 18 to 61 years; *M* = 24.81, *SD* = 8.23) who had experienced trauma meeting PTSD Criterion A as defined by the Diagnostic and Statistical Manual of Mental Disorders, Fifth Edition (DSM‐5; American Psychiatric Association 2013) were recruited through community advertisements and psychiatric outpatient clinics at a medical center in central Taiwan (background variables presented in Table 1). Participants were excluded if they (1) had a current or past history of general medical illnesses or neurological conditions that could interfere with cognitive function or (2) lacked adequate communication or language skills to understand the study instructions. Based on their most distressing traumatic event, participants were categorized into either the interpersonal trauma group (*n* = 72) or the impersonal trauma group (*n* = 128). While there was no significant age difference between the groups (*t*(198) = 1.53, *p* = 0.128), a significant gender difference was found (*χ* ^*2*^(1) = 7.51, *p* = 0.006), with a higher proportion of women in the interpersonal trauma group.

**TABLE 1:** Background variables of study participants (*N* = 200).

Variables	Level	Number	Percentage (%)
Gender	Men	69	34.5
	Women	131	65.5
Education level	High school/fifth junior	25	12.3
	College/university	162	80.5
	Master	14	7.0
Marital status	Unmarried	182	91.0
	Married	12	6.0
	Divorce	5	2.65
	Widowed	1	0.5
Profession	Student	145	72.5
	Service industry	24	12.0
	Education industry	10	5.0
	Other	21	10.5
The most disturbing traumatic event	**Interpersonal traumas**	**72**	**36.0**
	Physical assault	15	7.5
	Assault with a weapon	2	1.0
	Sexual assault	7	3.5
	Other unwanted or uncomfortable sexual experiences	8	4.0
	Others, such as bullying	40	0.2
	**Non‐interpersonal traumas**	**128**	**64.0**
	Natural disaster	17	8.5
	Fire or explosion	2	1.0
	Transportation accident	28	14.0
	Serious accident	6	3.0
	Exposure to toxic substances	1	0.5
	War or exposure to a war zone	3	1.5
	Life‐threatening illness or injury	24	12.0
	Severe human suffering	2	1.0
	Sudden violent death	4	2.0
	Sudden unexpected death	9	4.5
	Causing serious injury, harm, or death to another person	2	1.0
	Others: witnessing a car accident	30	15.0

A two‐wave longitudinal design captured temporal changes between PTSS and PAU. Of the 200 traumatized individuals who completed the initial assessment (time 1; T1) 143 completed follow‐up assessments at one month (time 2; T2). All missing data at T2 were excluded from the analyses. Note that there was no difference in age (*t*(198) = 0.57, *p* = 0.571), EF(*t*(198) = 0.97, *p* = 0.333), DANVA (*t*(198) = −0.44, *p* = 0.662), cognitive and affective ToM (*t*(198) = 1.26, *p* = 0.210; *t*(198) = 0.57, *p* = 0.571) and AUDIT score (*t*(198) = −1.33, *p* = 0.001), but significant differences in gender (*χ* ^*2*^(1) = 9.46, *p* = 0.002) and PTSS score (*t*(111.938) = 3.42, *p* = 0.001) between individuals who did (*n* = 143) and did not (*n* = 57) complete a one‐month follow‐up, indicating women with higher PDS total scores were more likely to complete the follow‐up. Gender was therefore controlled as a potential confounding variable in subsequent analyses. Moreover, the elevated PTSS scores confirm that the current sample is representative of a trauma‐exposed population.

### 3.3 Instruments

#### 3.3.1 MASC‐TW

The MASC, originally developed by Dziobek et al. in 2006, is an English‐language, video‐based test designed to assess cognitive and affective ToM. The test features a 15‐minute video depicting a social dinner scene with two men and two women, illustrating social situations involving misunderstandings, irony, body language, ambiguity, flirting and insults. The MASC is presented to participants via PowerPoint, with the video paused 45 times. At each pause, participants are asked a multiple‐choice question that requires them to infer the thoughts, feelings or intentions of the characters. Example questions include "Why do you think Betty made this comment?" and "How is Michael feeling?" To respond, participants must interpret verbal cues (required for 19 items, including 10 literal and 6 nonliteral) and nonverbal cues (required for 16 items, including 6 related to facial expressions and 10 related to other nonverbal cues). Each question provides four response options, with one correct answer reflecting the accurate attribution of ToM to the characters. Overall performance is measured by the total score of correct answers, with a maximum score of 45. The MASC also calculates two subscale scores: cognitive ToM (26 items, maximum score = 26) and affective ToM (18 items, maximum score = 18). The Taiwanese version of the MASC (MASC‐TW; Huang et al. 2023) is the Mandarin adaptation of the original English MASC. This version has demonstrated strong reliability, with a high Cronbach's α of 0.87 and a high intraclass correlation coefficient of 0.85 over time.

#### 3.3.2 DANVA‐2‐TW.

The DANVA‐2‐TW (Chen 2006) is a validated and culturally adapted nonverbal assessment tool designed for the Han Chinese population in Taiwan with satisfactory inter‐rater and test‐retest reliability (Tseng et al. 2012, 2013). This instrument has demonstrated strong interrater and test‐retest reliability. The present study utilized 48 facial photographs and 48 vocal clips to assess facial and prosodic emotion recognition, each representing one of four basic emotions—happiness, sadness, anger and fearfulness (12 items per emotion category)—from the DANVA‐2‐TW. Each photograph and vocal clip were categorized with at least 80% agreement on the intended emotion. Photographs were displayed on a laptop screen in full‐screen mode (resolution: 1024 × 768) for 500 milliseconds, while vocal clips were played through earphones, lasting 2 to 5 seconds. After viewing or listening to each stimulus, participants were required to make a forced choice among the four emotion categories. Accuracy for each emotion category was measured as the proportion of correct responses, with scores ranging from 0% (entirely incorrect) to 100% (entirely correct). This study applied a correction method based on Wagner's approach to minimize response bias. Wagner's (1993) unbiased hit rate (Hu) was calculated as the product of detection probability and hit frequency, using the formula: Hu = (Ai/Bi) × (Ai/Ci), where Ai represents the frequency of hits, Bi is the number of trials in which i is the target, and Ci denotes the frequency of responses categorized as i.

#### 3.3.3 WCST

A computerized version of the Wisconsin Card Sorting Test‐64 (WCST‐64; Heaton 1981) was utilized to evaluate participants' ability to adapt to shifting reinforcement patterns. Participants matched 64 response cards to one of four stimulus cards based on three possible dimensions: colour, form or number. Selections were made by pressing one of four designated number keys on a computer keyboard. The WCST provided four primary scores: number of categories correct, number of perseverative errors, number of total correct and percent of perseverative errors. For this study, the total number of correct responses was used as the index of EF.

#### 3.3.4 Traumatic Life Events

Traumatic life events were assessed by using the Life Event Checklist for DSM‐5 (LEC‐5; Gray et al. 2004; Weathers et al. 2013) to assess potentially traumatic life events and are based on PTSD Criteria A in the DSM‐5. This measure includes 17 potentially traumatic life events. When completing this questionnaire, participants were asked to select the most distressing traumatic event they had experienced. The severity of the PTSD symptoms toward the most distressing traumatic event was assessed by the Posttraumatic Diagnostic Scale for DSM‐5.

#### 3.3.5 Posttraumatic Stress Symptoms

The Posttraumatic Diagnostic Scale for DSM‐5 (PDS‐5; Foa et al. 2016) is a self‐report instrument designed to assess posttraumatic stress disorder (PTSD) based on the diagnostic criteria outlined in the DSM‐5 (American Psychiatric Association 2013). The scale employs a five‐point Likert scale ranging from 0 (not at all) to 4 (six or more times a week/severe). It consists of 26 items: two items screen for traumatic events, 20 items assess PTSD symptom severity across four domains (intrusion, avoidance, changes in mood and cognition, and arousal/hyperreactivity), two items evaluate the distress caused by symptoms, and two items measure the onset and duration of symptoms. Higher scores indicate greater PTSD symptom severity. The Chinese version of the PDS‐5 with excellent internal consistency and satisfactory temporal stability over five weeks and one year across diverse trauma types (Su et al. 2020) was used. In the present study, the Chinese PDS‐5 exhibited good internal consistency, with Cronbach's α values of 0.91, 0.80, 0.91 and 0.85 for the subscales of intrusion, avoidance, changes in mood and cognition, and arousal/hyperreactivity, respectively and an overall α = 0.96 for the total score.

#### 3.3.6 AUDIT

The Alcohol Use Disorders Identification Test (AUDIT) is a 10‐item screening instrument developed by the World Health Organization (WHO) (Saunders et al. 1993) to evaluate alcohol consumption, drinking behaviours and alcohol‐related problems. It includes both a clinician‐administered version and a self‐report version and we used the self‐report version. Patients are encouraged to respond to AUDIT questions using standard drink measurements, with a reference chart provided to illustrate the approximate number of standard drinks in various alcoholic beverages. A score of 8 or higher indicates hazardous or harmful alcohol use. The Chinese version of the AUDIT has been validated in a hospitalized Taiwanese population as a result of good reliability and validity (Tsai et al. 2005). A two‐factor solution for the AUDIT (alcohol consumption and alcohol‐related consequences) was proposed (Doyle et al. 2007). Alcohol‐related consequences were chosen as the index of PAU because a cross‐country study found that the AUDIT alcohol problems subscale, compared with the alcohol consumption subscale, exhibited smaller overall differences across countries and language subgroups (Horváth et al. 2023). In the present study, the Chinese AUDIT exhibited good internal consistency, with Cronbach's α values of 0.83, 0.84 and 0.89 for the two subscales and the total score, respectively.

### 3.4 Statistical Analysis

Data analyses were performed using SPSS, version 29.0. Pearson or Spearman correlations were used to assess the associations between PTSS, PAU and other study variables over time. Then, a path analysis of the association between PTSS, PAU, executive function, emotion recognition and ToM was conducted using Amos 16.0. Model fit was evaluated based on several indicators: 1) a chi‐square statistic (χ^2^), 2) the comparative fit index (CFI), and 3) the root mean square error of approximation (RMSEA) with a 90% confidence interval, and 4) SRMR (Standardized Root Mean Square Residual). For model fit evaluation, a nonsignificant chi‐square statistic, CFI ≥ 0.95, RMSEA ≤ 0.06 and SRMR ≤ 0.05 were considered to indicate excellent model fit, and a nonsignificant chi‐square statistic, CFI ≥ 0.90, RMSEA ≤ 0.08 and SRMR ≤ 0.08 were considered to indicate adequate model fit. The bootstrapping maximum likelihood estimator (5,000 resamples) method with bias‐corrected 95% confidence intervals (CIs) was employed to investigate direct and indirect effects. All effects were significant when the 95% CI did not contain zero. Data will be made available on OSF (https://osf.io/sntg3/files/osfstorage/68b14bc83ad3c6aadaab15c1).

## 4 Results

### 4.1 Descriptive Statistics

Table 2 presents the mean, standard deviation, skewness and kurtosis for each variable. Curran et al. (1996) suggest that, when assessing multivariate normality, if the absolute value of the skewness coefficient is below 2 and the absolute value of the kurtosis coefficient is below 7, data are considered generally distributed. In this study, most skewness coefficients are under 2, and all kurtosis coefficients are below 7, except for AUDIT scores. The test of multivariate normality was also violated with a critical value of 1.96 for Mardia's coefficient (= 15.69 at T1, 21.44 at T2), indicating a non‐normal distribution. The bootstrapping maximum likelihood estimator was recommended under non‐normal data conditions in a large sample size. The bootstrapping maximum likelihood estimator was recommended under non‐normal data conditions in a large sample size.

**TABLE 2:** Descriptive statistics for all study variables at Time 1 (*N* = 200).

Variable	Mean	SD	Skewness	Kurtosis
**Age**	24.81	8.23	2.17	4.54
**PDS total score**	20.67	17.51	0.64	−0.61
Intrusion	5.70	4.74	0.56	−0.49
Avoidance	2.63	2.40	0.50	−0.95
Changes in mood and cognition	7.16	6.88	0.82	−0.43
Arousal/hyperreactivity	5.19	5.11	0.95	0.25
**AUDIT total score**	5.55	6.56	2.18	5.91
Alcohol consumption	2.72	2.75	1.63	2.62
Alcohol consequence	2.83	4.33	2.42	7.34
**MASC‐TW correct score**	17.09	3.66	−1.77	5.90
Cognitive MASC‐TW	9.47	2.27	−1.40	4.45
Affective MASC‐TW	7.22	2.13	−1.01	1.45
**DANVA total accuracy**	0.69	0.11	−1.20	2.64
Facial emotion accuracy	0.66	0.12	−0.65	0.37
Prosodic emotion accuracy	0.72	0.15	−1.45	3.95
**WCST correct numbers**	47.10	9.01	−1.19	0.84

Abbreviations: AUDIT = the Alcohol Use Disorders Identification Test; DANVA = the Taiwanese version of the Diagnostic Analysis of Nonverbal Accuracy 2; MASC‐TW = the Taiwanese version of the A Movie for the Assessment of Social Cognition; PDS = the Posttraumatic Diagnostic Scale for DSM‐5; WCST = the Wisconsin Card Sorting Test‐64.

### 4.2 Associations Between PTSS, PAU, Executive Function, Emotion Recognition and ToM

Table 3 presents the correlation analyses across one month, and the results revealed significant correlations between gender and PDS total score and DANVA correct accuracy, as well as between age and the scores of AUDIT, MASC‐TW, DANVA and WCST, so gender and age were controlled in the following analyses. At Time1 and Time 2, PDS total score was positively associated with AUDIT alcohol consequence score, which supports the hypothesis (1) PTSS is positively associated with PAU across time. PDS total scores at Time 1 and Time 2 are associated with lower DANVA correct accuracy and lower WCST correct number, while alcohol consequence scores at Time 1 are associated with lower DANVA correct accuracy, lower cognitive and affective MASC‐TW scores, and lower WCST correct number; in addition, alcohol consequence scores at Time 2 were associated with lower cognitive and affective MASC‐TW scores. These results partially support the hypothesis (2) that executive function deficits and social cognition impairments are positively associated with PTSS and PAU across time. WCST correct numbers were significantly correlated with cognitive and affective MASC‐TW scores and DANVA correct accuracy, indicating that executive function and social cognition are interrelated.

**TABLE 3:** Correlations between all study variables.

Time1 (*N* = 200)	1	2	3	4	5	6	7	8	9
1. Gender.T1	—	—	—	—	—	—	—		
2. Age.T1	0.18*	—	—	—	—	—	—		
3. Cognitive MASC‐TW.T1	0.06	−0.30***	—	—	—	—	—		
4. Affective MASC‐TW.T1	−0.09	−0.32***	0.47***	—	—	—	—		
5. DANVA correct accuracy. T1	−0.21**	−0.24***	0.24**	0.45**	—	—	—		
6. WCST correct numbers. T1	0.02	−0.34***	0.29***	0.36***	0.36***	—	—		
7. PDS total score. T1	−0.23***	0.10	−0.11	−0.09	−0.15*	−0.20**	—		
8. AUDIT alcohol consequence. T1	0.04	0.37***	−0.23**	−0.29***	−0.15*	−0.17*	0.22**		
**Time2 (*N* = 143)**	**1**	**2**	**3**	**4**	**5**	**6**	**7**	**8**	**9**
9 .PDS total score. T2	−0.19*	0.14	−0.12	−0.14	−0.24**	−0.24**	0.81***	0.30**	—
10. AUDIT alcohol consequence. T2	0.07	0.33***	−0.16	−0.22**	−0.14	−0.16	0.18*	0.84***	0.28**

*Note:* T1 = Time1; T2 = Time2.

Abbreviations: AUDIT = the Alcohol Use Disorders Identification Test; DANVA = the Taiwanese version of the Diagnostic Analysis of Nonverbal Accuracy 2; MASC‐TW = the Taiwanese version of the A Movie for the Assessment of Social Cognition; PDS = the Posttraumatic Diagnostic Scale for DSM‐5; WCST = the Wisconsin Card Sorting Test‐64.

*p < 0.05; **p < 0.01; ***p < 0.001.

### 4.3 A Path Analysis of the Association Between PTSS, PAU, Executive Function, Emotion Recognition and ToM

To further investigate the correlation between PTSS, PAU, executive function, emotion recognition and social cognition over time, this study tested a hypothesized mediation model using Amos 16.0. The model included a pathway from PTSS (PDS total scores) to PAU (alcohol consequence scores) serially through executive function (WCST scores), emotion recognition (DANVA scores) and cognitive and affective ToM (MASC‐TW scores) at Time 1 and Time 2.

**FIGURE 1:**
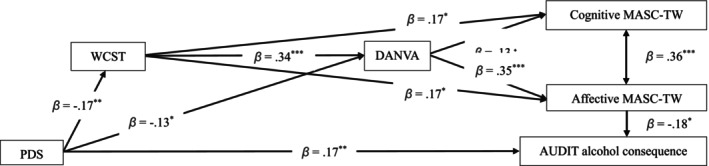
Mediation model at T1 after controlling for gender and age. AUDIT = the Alcohol Use Disorders Identification Test; DANVA = the Taiwanese version of the Diagnostic Analysis of Nonverbal Accuracy 2; MASC‐TW = the Taiwanese version of the A Movie for the Assessment of Social Cognition; PDS = the Posttraumatic Diagnostic Scale for DSM‐5; WCST = the Wisconsin Card Sorting Test‐64.^﹢^ *p* = 0.068; *p < 0.05; **p < 0.01; ***p < 0.001.

**FIGURE 2:**
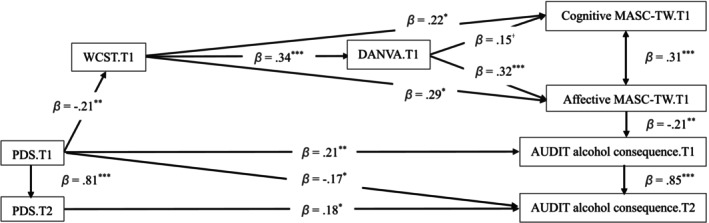
Mediation model from T1 to T2 after controlling for gender and age. AUDIT = the Alcohol Use Disorders Identification Test; DANVA = the Taiwanese version of the Diagnostic Analysis of Nonverbal Accuracy 2; MASC‐TW = the Taiwanese version of the A Movie for the Assessment of Social Cognition; PDS = the Posttraumatic Diagnostic Scale for DSM‐5; WCST = the Wisconsin Card Sorting Test‐64. T1 = Time1; T2 = Time2. ^﹢^ *p* = 0.079; *p < 0.05; **p < 0.01; ***p < 0.001.

After controlling for gender and age, both the model at T1 [*χ* ^*2*^ (10, *N* = 200) = 0.69, *p* = 0.731, CFI = 1.000, RMSEA = 0.000, 90% CI (0.057, 0.926), SRMR = 0.028; Figure 1] and the model from T1 to T2 [χ^2^ (25, *n* = 143) = 0.957, *p* = 0.524, CFI = 1.000, RMSEA = 0.000, 90% CI (0.000, 0.102), SRMR = 0.048; Figure 2] revealed excellent model fit to the data. Table 4 presents both models' results, including total, direct and indirect effects for each path. A bias‐corrected bootstrapping procedure with 95% confidence intervals was employed to ascertain the significance of these indirect effects.

**TABLE 4:** Direct and indirect effects of the path from PTSS to PAU after controlling for gender and age.

	AUDIT alcohol consequence. T1		
Total effects	Estimates	Lower 95% CI	Upper 95% CI
PDS ‐> WCST	−0.172	−0.263	−0.025
PDS ‐> DANVA	−0.190	−0.301	−0.040
PDS ‐> Cognitive MASC‐TW	−0.054	−0.100	−0.019
PDS ‐> Affective MASC‐TW	−0.096	−0.136	−0.034
PDS ‐> AUDIT	0.187	0.086	0.321
WCST ‐> DANVA	0.336	0.206	0.437
WCST ‐> Cognitive MASC‐TW	0.215	0.074	0.334
WCST ‐> Affective MASC‐TW	0.290	0.144	0.390
WCST ‐> AUDIT	−0.053	−0.111	−0.016
DANVA ‐> Cognitive MASC‐TW	0.129	−0.015	0.279
DANVA ‐> Affective MASC‐TW	0.349	0.227	0.468
DANVA ‐> AUDIT	−0.064	−0.122	−0.012
Affective MASC‐TW ‐> AUDIT	−0.184	−0.322	−0.029
**Direct effects**	**Estimates**	**Lower 95% CI**	**Upper 95% CI**
PDS ‐> WCST	−0.172	−0.263	−0.025
PDS ‐> DANVA	−0.132	−0.266	−0.002
PDS ‐> AUDIT	0.170	0.067	0.305
WCST ‐> DANVA	0.336	0.206	0.437
WCST ‐> Cognitive MASC‐TW	0.233	0.027	0.324
WCST ‐> Affective MASC‐TW	0.224	0.023	0.286
DANVA ‐> Cognitive MASC‐TW	0.156	−0.015	0.279
DANVA ‐> Affective MASC‐TW	0.370	0.227	0.468
Affective MASC‐TW ‐> AUDIT	−0.186	−0.322	−0.029
**Indirect effects**	**Estimates**	**Lower 95%CI**	**Upper 95%CI**
PDS ‐> DANVA via WCST	−0.058	−0.112	−0.015
PDS ‐> Cognitive MASC‐TW via WCST & DANVA	−0.054	0.001	−0.019
PDS ‐> Affective MASC‐TW via WCST & DANVA	−0.096	−0.141	−0.034
PDS ‐> AUDIT via WCST & DANVA & MASC‐TW	0.018	0.003	0.039
WCST ‐> Cognitive MASC‐TW via DANVA	0.043	0.001	0.132
WCST ‐> Affective MASC‐TW via DANVA	0.117	0.056	0.186
WCST ‐> AUDIT via DANVA & MASC‐TW	−0.053	−0.111	−0.016
DANVA ‐> AUDIT via MASC‐TW	0.064	−0.122	−0.012

	AUDIT alcohol consequence. T1 & T2		
Total effects	Estimates	Lower 95% CI	Upper 95% CI
PDS.T1 ‐> WCST.T1	−0.209	−0.341	−0.074
PDS.T1 ‐> DANVA.T1	−0.072	−0.143	−0.023
PDS.T1 ‐> Cognitive MASC‐TW.T1	−0.056	−0.120	−0.018
PDS.T1 ‐> Affective MASC‐TW.T1	−0.083	−0.161	−0.029
PDS.T1 ‐> PDS.T2	0.813	0.752	0.860
PDS.T1 ‐> AUDIT.T1	0.224	0.086	0.350
PDS.T1 ‐> AUDIT.T2	0.168	0.022	0.329
PDS.T2 ‐> AUDIT.T2	0.176	0.005	0.343
AUDIT.T1 ‐> AUDIT.T2	0.854	0.787	0.900
WCST.T1 ‐> DANVA.T1	0.343	0.215	0.464
WCST.T1 ‐> Cognitive MASC‐TW.T1	0.270	0.089	0.420
WCST.T1 ‐> Affective MASC‐TW.T1	0.398	0.234	0.543
WCST.T1 ‐> AUDIT.T1	−0.080	−0.173	−0.023
WCST.T1 ‐> AUDIT.T2	−0.068	−0.150	−0.020
DANVA.T1 ‐> Cognitive MASC‐TW.T1	0.145	−0.017	0.303
DANVA.T1 ‐> Affective MASC‐TW.T1	0.318	0.169	0.450
DANVA.T1 ‐> AUDIT.T1	−0.064	−0.147	−0.016
DANVA.T1 ‐> AUDIT.T2	−0.054	−0.129	−0.014
Affective MASC‐TW.T1 ‐> AUDIT.T1	−0.200	−0.361	−0.056
Affective MASC‐TW.T1 ‐> AUDIT.T2	−0.171	−0.316	−0.048
**Direct effects**	**Estimates**	**Lower 95% CI**	**Upper 95% CI**
PDS.T1 ‐> WCST.T1	−0.209	−0.341	−0.074
PDS.T1 ‐> PDS.T2	0.813	0.752	0.860
PDS.T1 ‐> AUDIT.T1	0.207	0.069	0.336
PDS.T1 ‐> AUDIT.T2	−0.166	−0.329	0.004
PDS.T2 ‐> AUDIT.T2	0.176	0.005	0.343
WCST.T1 ‐> DANVA.T1	0.343	0.215	0.464
WCST.T1 ‐> Cognitive MASC‐TW.T1	0.221	0.030	0.387
WCST.T1 ‐> Affective MASC‐TW.T1	0.289	0.122	0.452
DANVA.T1 ‐> Cognitive MASC‐TW.T1	0.145	−0.017	0.303
DANVA.T1 ‐> Affective MASC‐TW.T1	0.318	0.169	0.450
Affective MASC‐TW.T1 ‐> AUDIT.T1	−0.200	−0.361	−0.056
**Indirect effects**	**Estimates**	**Lower 95% CI**	**Upper 95% CI**
PDS.T1 ‐> DANVA.T1 via WCST.T1	−0.072	−0.143	−0.023
PDS.T1 ‐> Cognitive MASC‐TW.T1 via WCST.T1 & DANVA.T1	−0.056	−0.120	−0.018
PDS.T1 ‐> Affective MASC‐TW.T1 via WCST.T1 & DANVA.T1	−0.083	−0.161	−0.029
PDS.T1 ‐> AUDIT.T1 via WCST.T1 & DANVA.T1 & MASC‐TW.T1	0.017	0.004	0.049
PDS.T1 ‐> AUDIT.T2 via WCST.T1 & DANVA.T1 & MASC‐TW.T1	0.334	0.180	0.512
WCST.T1 ‐> Cognitive MASC‐TW.T1 via DANVA.T1	0.050	0.000	0.129
WCST.T1 ‐> Affective MASC‐TW.T1 via DANVA.T1	0.109	0.058	0.184
WCST.T1 ‐> AUDIT.T1 via DANVA.T1 & MASC‐TW.T1	−0.080	−0.173	−0.023
WCST .T1‐> AUDIT.T2 via DANVA.T1 & MASC‐TW.T1	−0.068	−0.150	−0.020
DANVA.T1 ‐> AUDIT.T1 via MASC‐TW.T1	−0.064	−0.147	−0.016
DANVA.T1 ‐> AUDIT.T2 via MASC‐TW.T1	−0.054	−0.129	−0.014
Affective MASC‐TW.T1 ‐> AUDIT.T2 via AUDIT.T1	−0.171	−0.316	−0.048

*Note:* T1 = Time1; T2 = Time2.

Abbreviations: AUDIT = the Alcohol Use Disorders Identification Test; DANVA = the Taiwanese version of the Diagnostic Analysis of Nonverbal Accuracy 2; MASC‐TW = the Taiwanese version of the A Movie for the Assessment of Social Cognition; PDS = the Posttraumatic Diagnostic Scale for DSM‐5; WCST = the Wisconsin Card Sorting Test‐64.

At Time 1 (Table 4, Figure 1), all direct paths were significant except for the path from PDS total scores at T1 to AUDIT alcohol consequence scores at T2. Similarly, all indirect paths were significant except for the pathway from PDS to cognitive MASC‐TW scores through WCST and DANVA scores. From Time 1 to Time 2 (Table 4, Figure 2), all direct paths were significant except for the path from DANVA scores at T1 to cognitive MASC‐TW scores at T1, although several significant direct pathways emerged. However, one indirect pathway from T1 to T2 was nonsignificant, namely the path from WCST scores to cognitive MASC‐TW scores through DANVA scores. Overall, these findings indicate that PTSS not only directly predicts PAU but also indirectly contributes to PAU via deficits in EF, emotion recognition and ToM. These results partially support the hypothesis (3) that EF deficits and social cognition impairments sequentially mediate the relationship between PTSS and PAU across time.

## 5 Discussion

This study examined whether cognitive deficits in executive function (EF) and social cognition impairments (emotion recognition, cognitive ToM and affective ToM) mediate the relationship between posttraumatic stress symptoms (PTSS) and problematic alcohol use (PAU). The findings showed that PTSS not only directly predicted PAU but also indirectly influenced it through impairments in EF, emotion recognition and ToM, lending support to the self‐medication hypothesis.

Our first finding showed that PTSS at Time 1 (T1) directly predicted PAU at T1 and indirectly predicted PAU at Time 2 (T2), supporting the drinking‐to‐cope self‐medication model. According to Khantzian (1997), individuals with mental health difficulties may use substances such as alcohol to reduce psychological distress, representing an adaptive but maladaptive coping strategy. Applied to trauma, this perspective suggests that survivors often use alcohol to alleviate PTSS, thereby reinforcing negative reinforcement learning (Hawn et al. 2020). Although alcohol provides temporary relief, repeated use to manage trauma‐related distress can lead to dependence and, over time, escalate into problematic alcohol use or alcohol use disorders. In this cycle, alcohol both alleviates and sustains PTSS: while it helps individuals avoid negative emotions, it simultaneously interferes with trauma recovery and perpetuates symptoms. This reciprocal process explains the strong observed relationship between PTSS at T1 and PAU across both T1 and T2.

However, Hawn et al. (2020) emphasized that trauma survivors do not respond to distress uniformly. While some individuals turn to alcohol or drugs as coping mechanisms, others rely on avoidance or emotional numbing, which may lead to social withdrawal and reduced alcohol use. This variability may explain why PTSS at T1 negatively predicted PAU at T2. Moreover, examination of mean PTSS scores revealed a decline from T1 (*M* = 23.17) to T2 (*M* = 20.24), suggesting a general reduction in symptoms over time. Such decreases in PTSS could contribute to lower alcohol consumption at follow‐up. Future research employing longer follow‐up periods is needed to clarify the temporal dynamics between PTSS and PAU.

Our second hypothesis, that PTSS would be associated with impairments in EF, emotion recognition and cognitive and affective ToM, was partially supported. As shown in Figures 1 and 2, trauma was linked to reduced EF and disrupted social cognition, with affective ToM further contributing to problematic alcohol use (PAU). These findings are consistent with previous research indicating that trauma adversely impacts brain regions involved in stress regulation, including the amygdala, hippocampus and prefrontal cortex (Bremner 2006). Damage to these areas may underlie observed deficits in EF, emotion recognition and ToM, thereby increasing vulnerability to maladaptive coping strategies such as alcohol use.

In line with our findings, PAU has been consistently associated with impairments in both executive and social cognition. Evidence shows that individuals with PAU exhibit deficits in a broad range of cognitive functions, including IQ, verbal fluency, processing speed, working memory, attention, executive control, verbal and visual learning, memory and visuospatial abilities (Stavro et al. 2013). Social cognition is also impaired, with difficulties in expressing and decoding emotions, reduced ability to infer others' mental states (ToM deficits), and diminished empathy performance. Such cognitive and social‐cognitive impairments are considered key risk factors for PAU, contributing not only to its development but also to its persistence.

Our third hypothesis, that PTSS indirectly predicts PAU through impairments in EF, emotion recognition and ToM, was supported. EF and emotion recognition played central roles in shaping ToM, with a pathway linking EF to both cognitive and affective ToM via emotion recognition. This aligns with the two‐system framework of ToM (Meinhardt‐Injac et al. 2018), which posits that understanding others' mental states involves both explicit cognitive control and implicit social‐perceptual processes. In this context, EF provides the cognitive resources needed for perspective‐taking, while emotion recognition facilitates the accurate interpretation of social cues, jointly supporting ToM.

Our findings also resonate with the I‐PACE (Interaction of Person–Affect–Cognition–Execution) model of addictive behaviours (Brand et al. 2016). This framework emphasizes the dynamic interplay of personality traits (Person), emotional states (Affect), cognitive processes (Cognition) and executive functioning (Execution) in the development and maintenance of addictions. In our study, posttraumatic stress responses reflect affective dysregulation (Affect), social cognition abilities represent cognitive processing (Cognition), and decision‐making assessed by the WCST captures executive control (Execution). When trauma‐exposed individuals experience persistent negative emotions and misinterpret others' intentions, this negative bias may heighten sensitivity to trauma‐related cues and impair both EF and social cognition. With diminished emotional self‐regulation, individuals may increasingly rely on alcohol as a coping strategy, thereby reinforcing the cycle of distress and alcohol misuse. Future research should extend this framework by including additional variables, such as personality traits or resilience factors, to further validate and refine the I‐PACE model in trauma‐related alcohol use.

Notably, only affective ToM significantly predicted PAU in this study. One possible explanation is that both affective ToM and alcohol misuse are associated with overlapping structural and functional alterations in brain regions such as the prefrontal cortex, amygdala and mirror neuron system (MNS) (D'Hondt et al. 2014; Goldstein and Volkow 2011; Koob and Volkow 2016). These neural changes contribute to difficulties in emotional regulation and interpreting others' emotions, which may increase psychological distress and promote alcohol use as a self‐medication strategy (Khantzian 1997).

The findings also suggest that interventions for trauma‐exposed individuals with alcohol misuse should extend beyond craving management to address cognitive and social‐cognitive deficits. Strengthening EF through cognitive remediation (Rupp et al. 2012) and enhancing emotion recognition through social cognition training (Nandrino et al. 2021) may reduce reliance on alcohol for coping. Integrating these approaches into trauma‐focused treatments could provide a more comprehensive strategy to improve both trauma recovery and long‐term substance use outcomes.

Several limitations should be acknowledged. First, although participants were recruited from both the community and psychiatric outpatient clinics, the majority were students (72%), which limits the generalizability of the findings. Future research should therefore recruit more diverse samples to enhance representativeness. Second, the average PTSS and AUDIT scores were slightly below the respective clinical cut‐offs of 28 and 8, indicating that replication with clinical populations is needed to strengthen applicability. Third, the indirect effect of PTSS on PAU was relatively small, highlighting the need for larger samples and inclusion of variables such as age of onset to clarify the directionality of associations. Finally, not all study variables were reassessed at Time 2, and the follow‐up period was limited to one month, restricting causal inference. Future longitudinal studies with longer follow‐up intervals and repeated measurement of all variables would allow for a more comprehensive understanding of the dynamic relationships among trauma, cognition and alcohol use.

Despite these limitations, this study is among the first to provide prospective evidence that deficits in both general cognition and social cognition significantly influence the relationship between PTSS and PAU. These findings underscore the importance of incorporating cognitive and social‐cognitive components into interventions for trauma‐exposed individuals, as strengthening these abilities may help reduce alcohol misuse and promote more effective trauma recovery.

## 6 Conclusion

This study examined the mediating roles of executive function (EF) and social cognition (emotion recognition, cognitive ToM and affective ToM) in the relationship between posttraumatic stress symptoms (PTSS) and problematic alcohol use (PAU) using a two‐wave longitudinal design. The findings demonstrated that PTSS directly predicted PAU and also indirectly influenced PAU through impairments in EF and social cognition, supporting the self‐medication hypothesis. Importantly, affective ToM emerged as a unique predictor of PAU, highlighting the role of emotional understanding in trauma‐related alcohol misuse.

The results extend theoretical models, such as the two‐system framework of ToM and the I‐PACE model, by illustrating how trauma‐related cognitive and social‐cognitive deficits contribute to alcohol use as a maladaptive coping strategy. Clinically, the findings underscore the need for interventions that move beyond craving management to also target EF and social cognition. Cognitive remediation and social‐cognitive training, when integrated into trauma‐focused treatments, may reduce reliance on alcohol and improve long‐term recovery outcomes.

Although the study was limited by its predominantly student sample, modest clinical severity, small indirect effects and short follow‐up period, it provides novel evidence that both cognitive and social‐cognitive processes play key roles in the PTSS–PAU pathway. Future research should employ larger and more diverse samples, longer follow‐up intervals, and repeated measures of all variables to strengthen causal inference.

In sum, this study highlights the importance of considering cognitive and social‐cognitive functioning in understanding and addressing the comorbidity of PTSS and alcohol misuse. Targeting these mechanisms in prevention and intervention efforts may provide more comprehensive and effective strategies for supporting trauma‐exposed individuals.
